# Pharmacologic targeting of endothelial Ca^2+^-activated K^+^ channels: A strategy to improve cardiovascular function

**DOI:** 10.1080/19336950.2018.1454814

**Published:** 2018-04-16

**Authors:** Cini Mathew John, Rayan Khaddaj Mallat, Grace George, Taeyeob Kim, Ramesh C. Mishra, Andrew P. Braun

**Affiliations:** Department of Physiology and Pharmacology, Cumming School of Medicine, University of Calgary, Calgary, Alberta, Canada

**Keywords:** blood pressure, calcium-activated K^+^ channels, nitric oxide, endothelium, hyperpolarization, SKA-31, vasodilation

## Abstract

Endothelial small and intermediate-conductance, Ca^2+^-activated K^+^ channels (KCa2.3 and KCa3.1, respectively) play an important role in the regulation of vascular function and systemic blood pressure. Growing evidence indicates that they are intimately involved in agonist-evoked vasodilation of small resistance arteries throughout the circulation. Small molecule activators of KCa2.x and 3.1 channels, such as SKA-31, can acutely inhibit myogenic tone in isolated resistance arteries, induce effective vasodilation in intact vascular beds, such as the coronary circulation, and acutely decrease systemic blood pressure in vivo. The blood pressure-lowering effect of SKA-31, and early indications of improvement in endothelial dysfunction suggest that endothelial KCa channel activators could eventually be developed into a new class of endothelial targeted agents to combat hypertension or atherosclerosis. This review summarises recent insights into the activation of endothelial Ca^2+^ activated K^+^ channels in various vascular beds, and how tools, such as SKA-31, may be beneficial in disease-related conditions.

## Introduction

Endothelial small and intermediate conductance, Ca^2+^-activated K^+^ channels, commonly known as SKCa or KCa2.3, and IKCa or KCa3.1 channels, respectively, are widespread in the mammalian vasculature and increasingly implicated in various physiological mechanisms, including the regulation of vascular tone and membrane potential of small resistance arteries [1,2], and vascular remodelling [3]. There are four major classes of K^+^-selective channels that exhibit unique electrophysiological and pharmacological properties. These classes include: inwardly rectifying K^+^ channels (Kir channels); tandem pore domain K^+^ channels (K2P channel); Ca^2+^ activated K^+^ channels (KCa channels: BKCa, IKCa and SKCa) and voltage-gated K^+^ channels (Kv) [4]. This review focuses on Ca^2+^ activated K^+^ channels expressed in the endothelium (i.e. KCa2.3 and KCa3.1 channels), which are non-voltage gated, and open in response to elevated cytosolic free Ca^2+^. Under physiological conditions, activation of endothelial KCa channels leads to electrical transmission along the endothelial layer as well as electrical signalling between the endothelium and overlying vascular smooth muscle (i.e. endothelium-derived hyperpolarization, EDH) that can elicit vasodilation in resistance arteries by reducing L-type Ca^2+^ channel activity and Ca^2+^ entry in smooth muscle [5]. The activation of endothelial KCa channels by Ca^2+^ mobilizing, vasodilatory stimuli (e.g. acetylcholine) also contributes to *de novo* nitric oxide (NO) generation via enhanced Ca^2+^ elevation in the endothelium, which occurs via endothelial hyperpolarization and augmented Ca^2+^ influx [6] (Fig. 2). As described below, pharmacologic or genetically induced changes in the activity of endothelial KCa channels are associated with various pathological conditions, including cardiovascular [7], metabolic [5], and inflammatory diseases [8]. Translational research has further strengthened the involvement of KCa 2.x and KCa3.1 channels in various animal and human models of disease [5,9–11]. Research efforts to elucidate the functional roles of endothelial KCa2.3 and KCa3.1 channels have benefited tremendously from the availability and development of selective KCa channel pharmacologic agents. One of the earliest and best studied examples is apamin, an 18 amino acid peptide found in bee venom that blocks KCa2.x channels from the external surface with high potency (IC_50_ range 0.2-20 nM) [12]. Potent peptide inhibitors of KCa3.1 channels have also been isolated from scorpion venom (e.g. charybdotoxin, maurotoxin), however, these latter agents can also block subtypes of voltage-gated K^+^ channels, thereby limiting their use in mechanistic studies. The development and validation of TRAM-34 as a synthetic, highly selective, small molecule inhibitor of KCa3.1 channels has demonstrated that it is possible to selectively target KCa channels [13], and this work has led to the rational design of small molecules that act as endothelial KCa channel activators or potentiators [14]. For a more comprehensive discussion of KCa and Kv channel pharmacologic agents, the reader is referred to several detailed review articles [15–18]. Using the FDA-approved neuroprotectant agent riluzole (a non-selective activator of KCa2.x and KCa3.1 channels with micromolar efficacy) as a chemical template, Wulff and coworkers synthesized a series of novel compounds displaying differing degrees of activation and selectivity for KCa2.3 and KCa3.1 channels [19–21]. Of the various derivatives examined, SKA-31 has been widely utilized as a selective KCa channel activator, displaying approximately 10-fold greater potency for KCa3.1 channels (EC_50_ ∼ 0.2 µM) vs KCa2.3 (EC_50_ ∼ 2 µM), and little to no effect on various other voltage-gated cation channels. At the cellular level, endothelial KCa channel activators have been shown to enhance the stimulated production of endothelial NO [6], which would be expected to counteract endothelial dysfunction, and acute systemic administration of SKA-31 is reported to lower blood pressure in both rodents and large animal models [19,22,23]. In addition, treatment of isolated beating hearts from a rodent model of non-obese Type 2 Diabetes (T2D) (i.e. Goto-Kakizaki rats) with SKA-31 produced an immediate improvement in endothelium-dependent dilation and fluid flow in the coronary circulation [5]. In the following review, we will summarize a number of key observations from our group and others highlighting the effects of SKA-31 in the cardiovascular system under health and disease conditions.

## Calcium dependence of endothelial KCa channel activation

KCa2.x channels (i.e. KCa2.1, 2.2 and 2.3) are encoded by three separate, paralogous genes, which are distinct from the gene encoding the KCa3.1 channel [24]. The core region of KCa2.x channels (i.e. transmembrane segments S1 to S6) displays 80–90% predicted amino acid identity, with N- and C-terminal domains exhibiting more divergence [25]. By comparison, the KCa3.1 channel displays only 42–44% primary sequence identity with KCa2.x channels over the same S1-S6 core region [26]. Although KCa2.x and KCa3.1 channels exhibit clear differences with respect to their biophysical properties and pharmacologic sensitivities, they also share certain behavioural qualities. Principally, the activation of both KCa2.x and KCa3.1 channels is voltage-independent (i.e. absence of a functional voltage sensor domain), and instead, is reliant upon a Ca^2+^/calmodulin-mediated gating mechanism [27]. Calmodulin is a ubiquitously expressed, high affinity Ca^2+^ binding protein that plays an important role as an intracellular second messenger in modulating various Ca^2+^ signalling cascades [28].

Structurally, calmodulin is constitutively bound to an intracellular domain with KCa2.x and KCa3.1 channels, and effectively serves as an accessory subunit for these channels. The binding of cytosolic Ca^2+^ to channel-bound calmodulin leads to channel opening by induction of a conformational change in the channel's intracellular gating machinery. KCa2/3 activators are termed positive gating modulators as they can promote further opening of the channels, typically by “sensitizing” the channels to Ca^2+^ calmodulin-dependent activation (i.e. a leftward shift in the channel opening vs. free Ca^2+^ relation) [20]. Structure/function analyses and computational modeling of the KCa3.1 channel have identified the putative molecular binding site for SKA-31 and related KCa channel agonists (e.g. NS309, EBIO, SKA-121, SKA-111) to the interface between the channel's C-terminal calmodulin binding domain (CaM-BD) and bound calmodulin [21]. Amino acid residues contributing to key drug-protein interactions identified in the KCa3.1 channel closely agree with the binding site interactions described for crystal structures of the closely related KCa2.2 channel CaM-BD + calmodulin in the presence of either NS-309 or EBIO [29,30]. It is likely that the binding of KCa channel positive modulators at this protein interface facilitates/stabilizes the opening of KCa3.1 and KCa2.x channels by Ca^2+^-bound calmodulin, leading to the apparent increase in sensitivity of these channels to activation by cytosolic Ca^2+^.

The KCa3.1 channel is expressed in a variety of non-excitable cells, including erythrocytes (i.e. the Gardos channel), platelets, T and B lymphocytes, mast cells, monocytes/macrophages, microglia and vascular endothelial cells [31]. In many of these cells, KCa3.1 activity can augment Ca^2+^ influx in response to a stimulus (i.e. store-operated Ca^2+^ entry, SOCE) by increasing the electrical driving force for Ca^2+^ entry via membrane hyperpolarization. Following initial elevation of intracellular Ca^2+^ for example, following T cell receptor engagement in T lymphocytes, the membrane hyperpolarization generated by KCa3.1 channel opening enhances Ca^2+^ entry through cation-selective Orai channels [32].

## Structure activity relationship of SKA-31

**Figure 1.**
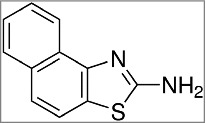
Chemical structure of SKA-31 (naphtho [1,2-*d*] thiazol-2-ylamine).

SKA-31 (naphtho[1,2-*d*]thiazol-2-ylamine) exhibits excellent pharmacokinetic properties such as a long half-life (12 h) and low plasma protein binding in rodents [19]. The EC_50_ values for SKA-31 mediated activation of KCa2.3 and KCa3.1 channels, measured electrophysiologically, are ∼2 μM and ∼0.2 µM, respectively. Based on structure-activity relationship studies, Coleman et al. (2014) reported that the continuous conjugation and replacement of the internal aromatic ring with an aliphatic group reduces the potency and selectivity of SKA-31 [20] (see Fig. 1). Furthermore, the amine group (-NH_2_) attached to the cyclopentene group is also required for its activity, which was verified by examining the effects of replacing the amine group with a methyl (-CH_3_) group. The sulphur atom of the cyclopentene ring also has certain significance in terms of activity, as the efficacy of SKA-31 was low in the absence of this sulphur atom. However, some actions of SKA-31 could be preserved if the sulphur atom was replaced with oxygen, which exhibits the same valence electrons [19,20].

**Figure 2.**
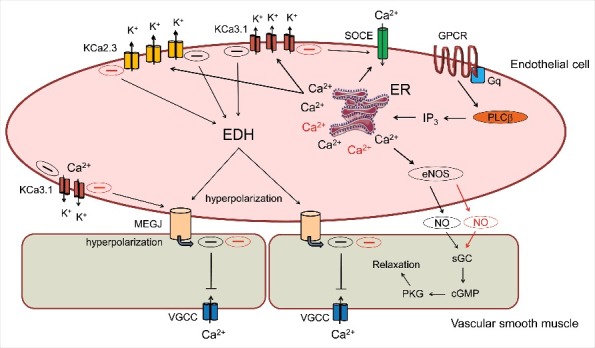
Cartoon depicting nitric oxide (NO) and hyperpolarization-dependent mechanisms contributing to agonist-induced vasodilation in resistance arteries. Stimulation of endothelial GPCR/Gαq complexes by Ca^2+^-mobilizing vasodilatory agonist promotes activation of PLC-β, the generation of IP_3_ via phosphatidyl inositol 4,5-bisphosphate (PIP_2_) hydrolysis and the opening IP3 receptors/Ca^2+^ release channels on the endoplasmic reticulum (ER). ER Ca^2+^ release is sensed by STIM1, an EF-hand protein localized in the ER membrane that migrates and couples with the Ca^2+^ influx channel Orai1, leading to Store-Operated Ca^2+^Entry (SOCE). The elevation of cytosolic Ca^2+^ by combined ER release and SOCE regulates the activity of endothelial NO synthase and Ca^2+^-activated K^+^ channels (KCa2.3 and KCa3.1); the latter effectors initiate endothelial-derived hyperpolarization (EDH) that can increase the electrical driving force for Ca^2+^ entry in endothelium and also transfer to the adjacent smooth muscle via myo-endothelial gap junctions (MEGJ) to reduce voltage-gated Ca^2+^ channel activity. The presence of SKA-31 or similar KCa channel positive modulators can pharmacologically “sensitize” KCa2.3 and KCa3.1 channel activation, thereby augmenting cell signaling mechanisms influenced by these channels. Key pathways that may be enhanced following KCa channel sensitization (i.e. generation and transfer of hyperpolarizing current, *de novo* NO generation) are highlighted with red shading.

## SKA-31 mediates endothelium-dependent effects

The vascular endothelium is a critical regulator of contractile tone in resistance arteries and thereby regulates vascular diameter, blood flow distribution and blood pressure [3,33–35]. The endothelium also contributes to the health of larger conduit arteries by limiting atherogenesis via nitric oxide signaling. Numerous functional observations highlight the pharmacological actions of SKA-31 in different tissues and animal models. Using arterial pressure myography, we demonstrated that SKA-31 evoked concentration-dependent inhibition of myogenic tone (i.e. increased intraluminal diameter) in rat cremaster and middle cerebral arteries [36] and mesenteric arteries (Khaddaj Mallat et al, unpublished data). This dilatory action of SKA-31 was prevented by mechanical disruption of the endothelium (i.e. passage of an air bubble through the vessel lumen), and in the presence of the KCa channel blockers TRAM-34 + UCL-1684, but not by exposure to the eNOS inhibitor L-NAME. These observations thus establish the endothelium as the major site of action for SKA-31 in small resistance arteries. In the vasculature, KCa3.1 appears to be the predominant channel involved in agonist (e.g. acetylcholine, ACh) evoked EDH responses and vasodilation of resistance arteries [37,38]. In contrast, KCa2.3 channels have been implicated more in flow-induced dilatory responses and active hyperaemia [39,40]. In both large conduit arteries and skeletal muscle cremaster resistance arteries isolated from genetically engineered mice with a defective KCa3.1 gene, the ACh-induced, EDH mediated vasodilatation was significantly reduced compared with responses in wild type mice [37]. Sankaranarayanan et al. (2009) have demonstrated that acute administration of SKA-31 in vivo decreases blood pressure of normotensive mice in a KCa3.1-dependent manner [19]. Similar effects were observed in short-term angiotensin II-induced hypertension in mice [19] and in conscious dogs after intravenous injection of SKA-31 [41]. We have also observed acute hypotensive actions of SKA-31 following intravenous administration in instrumented and anesthetized pigs [22]. Collectively, the activation of endothelial KCa3.1, along with KCa2.3 channels by SKA-31 and related compounds may represent a novel pharmacological strategy for lowering peripheral vascular resistance that may be beneficial in hypertension or ischaemic heart disease. The vascular EDH system can thus be considered as a powerful vasodilatory mechanism that acts in parallel to locally produced NO and prostacyclin (PGI2). Endothelial dysfunction is often characterized as a reduction in NO synthesis and/or NO bio-availability, and there is growing evidence that the EDH system is also affected in cardiovascular disease states, such as hypertension, diabetes, restenosis, chronic renal failure, and aging [42–44]. Although endothelial dysfunction is known to be an early clinical marker of more serious cardiovascular complications [45], it remains unclear whether endothelial dysfunction is a basic cause or a consequence of disease. In any case, components of the EDH system such as endothelial KCa2.3 and KCa3.1 channels may represent novel attractive targets for pharmacologic manipulation [46]. To date, however, no endothelial KCa channel activator/potentiator compounds have been approved for clinical use.

## Observed effects of SKA-31 in the periphery

In mammalian circulation, it is well known that small arteries (<300 micron diameter), arterioles and capillaries are mainly responsible for the regulation of systemic blood pressure and tissue blood flow. Accordingly, the presence of pathological conditions within this region of the vasculature can lead to disruption of blood pressure homeostasis [23]. SKA-31 is able to reduce mean arterial pressure in normotensive as well as in chronically hypertensive animals, and previous observations have provided insights into the time course of the systemic cardiovascular actions of SKA-31. In normo- and hypertensive mice, single intraperitoneal (I.P.) administrations of either 30 or 100 mg/kg SKA-31 reduced mean arterial pressure by 20–32 mmHg within 2 h. The time course of this SKA-31 mediated-hypotensive effect further agrees with the reported plasma concentrations of SKA-31, which appear to peak within 2 hours after a single I.P. injection [19]. Table 1 lists several studies in which the functional effects of SKA-31 in various tissues have been documented.

**Table 1.** Reported effects of SKA-31 in various tissues and animal models.

	Observed Functional Responses to SKA-31	Reference
1	Improvement of endothelium-dependent vasodilation	2, 5
2	Evoked hyperpolarization and decreased contractility in human urinary bladder smooth muscle	86, 87
3	Enhancement of agonist-evoked increases in coronary flow in the hearts of type 2 diabetic rats	5
4	Acute elevation of total coronary flow in male and female rat hearts	48
5	Potentiation of endothelium-derived hyperpolarization and lower blood pressure in rats	19
6	Enhancement of endothelial hyperpolarization and acute reduction of mean arterial blood pressure in conscious dogs	41
7	Acute increase in systemic vascular conductance and reduction in mean arterial pressure in anesthetized, instrumented pigs	22
8	Partial correction of abnormal Purkinje cell firing and improvement of motor function in SCA3 mice.	88

## Effects of SKA-31 in the coronary vasculature

In the coronary circulation, blood flow is regulated by both intrinsic (myogenic, endothelial) and extrinsic (hemodynamic and neural) mechanisms [47]. In isolated, Langendorff-perfused, spontaneously beating male and female rat hearts, basal coronary flow is reduced in the presence of the eNOS inhibitor L-NAME or the endothelial KCa channel blockers apamin + TRAM-34. In the presence of both treatments, coronary flow is further decreased in a synergistic manner [48]. These same inhibitors also impair coronary vasodilation in response to Ca^2+^ mobilizing, endothelium-dependent dilators such as acetylcholine and bradykinin. Importantly, bolus administration of SKA-31 acutely increases coronary flow in both male and female hearts, which is largely blocked in the presence of apamin + TRAM-34 [48]. Moreover, in isolated hearts from the Goto-Kakizaki non-obese T2D rat, which exhibits vascular endothelial dysfunction, SKA-31 evokes robust vasodilation of the coronary circulation that is not different from that observed in genetically matched, control animals [5]. We have further observed in anesthetized and instrumented juvenile pigs that intravenous administration of bolus SKA-31 (0.1-5 mg/kg) induces acute increases in coronary flow [22]. Direct intra-coronary administration of a related KCa channel activator (NS309) in anesthetized, open-chested dogs was also found to increase coronary flow, which was largely blocked in the presence of either TRAM-34 or the eNOS inhibitor L-NAME [49]. Functionally, blockade of coronary KCa3.1 channels with TRAM-34 did not impair reactive hyperemia in response to transient coronary occlusion in these same open-chested dogs [49]. In conscious, instrumented beagles, bolus administration of SKA-31 (2 mg/kg I.V.) produced a transient decrease in mean arterial pressure (∼ 25 mmHg) that was accompanied by a reflex tachycardia driven by peripheral baroreceptor activity [41]. Whereas KCa3.1 channels are largely absent from the mammalian myocardium, KCa2.2 channel expression and activity have been reported in atrial tissue, along with the sino-atrial and atrio-ventricular nodes from rodents and humans [50,51]. KCa2.x channel activity has further been associated with atrial fibrillation under pathophysiologic conditions, however, it has also been reported that KCa2.x channel inhibition can promote either anti-arrhythmic or pro-arrhythmic effects [52]. These mixed data may reflect differences in the species and/or model, severity and progression of cardiovascular disease. In anesthetized and instrumented juvenile pigs, we did not observe acute, pro-arrhythmic effects of the KCa channel activator SKA-31 at dosages producing significant decreases in mean arterial pressure [22], however, the effects of SKA-31 in an animal model of atrial fibrillation have yet to be reported.

## Role of SKA-31 in cerebral blood flow

The myogenic reactivity of cerebral resistance arteries is an important regulator of cerebral blood flow and is affected by vasodilatory signals originating within the endothelial layer [53]. In particular, KCa2.3 and KCa3.1 channels contribute to the modulation of basal tone in parenchymal cerebral arterioles; they induce smooth muscle hyperpolarization and enhance stimulated vasodilation [54]. MacNeish et al (2006) have demonstrated that KCa2.3 channels play a major role in mediating endothelial hyperpolarization [47] and another study has reported that the modulation of KCa2.3 and KCa3.1 channels in the endothelium of cerebral parenchymal arterioles has a substantial effect on cortical blood flow [55]. These observations suggest that dysfunction of endothelial KCa2.3 and KCa3.1 channels could compromise intra-cerebral blood flow (CBF), whereas activation of these channels is suggested to improve CBF [55].

## Beneficial effects of SKA-31 in vascular conditions characterised by endothelial dysfunction

### Cardiovascular diseases (CVD)

An early event often detected in the development/progression of CVD is injury or dysfunction of the vascular endothelium. As the primary interface between the blood and various tissues of the body, the vascular endothelium exhibits a diverse range of roles and activities, all of which contribute to the overall health and function of the cardiovascular system [56–58]. Thus, pathological conditions within the endothelium can lead to disruption of blood pressure homeostasis and tissue/organ perfusion. Activation of endothelial KCa2.3 and KCa3.1 channels is able to relax vascular smooth muscle tone, and may thus have therapeutic potential in select conditions (e.g. hypertension, atherosclerosis). In healthy adult rats, the KCa2.3 and KCa3.1 channel activator SKA-31 can evoke robust vasodilation in the coronary circulation in both male and female rat hearts [48]. In a non-obese rodent model of T2D exhibiting metabolic syndrome, we have further observed that SKA-31 dose-dependently increased total coronary flow to levels similar to that in control hearts, suggesting that endothelial KCa channel activity *per se* is not impaired in this model of T2D [5]. A previous study has demonstrated that SKA-31 and a related compound, SKA-20, could improve endothelial dysfunction in mice, and promote endothelium-dependent vasodilation in murine carotid arteries [2]. SKA-31 and similar reagents may therefore be useful for combination therapy in situations where existing antihypertensive drugs are not sufficiently effective or when it is considered desirable to increase blood flow in the microcirculation.

### Diabetes

T2D is becoming a global pandemic and attempts to mitigate endothelial dysfunction and its debilitating cardiovascular consequences in T2D patients have achieved only limited success, indicating that new, more effective strategies are needed. In light of the essential role played by endothelium-derived NO in the long-term function and health of the vasculature, it is logical that increasing the bioavailability of NO and vasodilatory capacity of the vasculature will oppose coronary artery disease, atherogenesis, hypertension and stroke in T2D patients exhibiting endothelial dysfunction. It is now well established that T2D and insulin resistance can cause endothelial dysfunction through a variety of mechanisms [59]. High levels of serum fatty acids and hyperglycaemia (i.e. metabolic syndrome), as seen in T2D patients, can increase oxidative stress, which can damage endothelial cells and limit eNOS activity and NO bioavailability [60]. Furthermore, it has been reported that a decrease in insulin action can result in a defective vasodilatory response [61]. Patients with T2D typically exhibit metabolic syndrome and endothelial dysfunction, which can lead to peripheral vasoconstriction and atherogenesis, making T2D a risk factor for cardiovascular disease (CVD), such as myocardial infarction. Improving endothelial function may be an effective therapeutic strategy for reducing the development/severity of CVD and atherosclerosis. Pharmacologic activators of endothelial KCa channels and enhancement of the EDH pathway may thus prove valuable in this regard [62].

### Obesity

Obesity is now recognized as an independent risk factor for CVD, and numerous investigations have revealed that there is a positive association between obesity and the initiation and progression of vascular dysfunction [63]. One of the diseases that can arise from such pathophysiological conditions is hypertension. Over the past two decades, obesity has become a major public health issue, as it increases the risk of developing cardiovascular and metabolic disease [64]. In healthy, young obese subjects, the clearance of triglyceride-rich lipoproteins is often impaired, and Danilo and Catapano (2007) have described evidence showing that triglyceride-rich lipoproteins may contribute significantly to the etiology of endothelial dysfunction and vascular disease [65]. In further support, studies have shown that endothelium-dependent vasodilation is reduced by 40–50% in obese patients when compared with lean patients [66]. These investigations involved measuring the differences in relative changes in leg blood flow in response to transient vasoconstriction (reactive hyperemia) or exercise-induced functional hyperemia. Collectively, these results suggest that increased levels of body fat, particularly visceral fat are associated with impaired endothelium-dependent vasodilation [67]. The association between endothelial dysfunction and increased blood pressure in obesity comes from studies showing that obese individuals display blunted vasodilatation in response to exogenously administered ACh in resistance arteries, as well as reduced capillary recruitment [68]. Moreover, the severity of endothelial dysfunction correlates with the degree of visceral adiposity [62]. Decreased NO bioavailability appears to play a major role in endothelial dysfunction in obesity-associated hypertension [69]. Functional uncoupling of endothelial nitric oxide synthase (i.e. enzymatic production of superoxide, O_2_^−^ instead of nitric oxide) may also contribute to decreased NO bioavailability [70], and such effects can be observed in an animal model of obesity without high blood pressure or hyperglycemia [71,72]. In coronary arteries from the obese, T2D Zucker rat, endothelial KCa2.3 and KCa3.1 channel activity and expression are upregulated and these vessels further exhibit an enhanced response to an established KCa channel activator (i.e. NS309) [73]. Obesity does not appear to disrupt endothelial KCa channel activity in other vascular beds [74], suggesting that maintenance and/or upregulation of endothelial KCa channel activity may help preserve stimulus-evoked vasodilation by opposing the development of endothelial dysfunction. Pharmacologic activators of endothelial KCa channels, such as SKA-31, may therefore prove beneficial by reducing the severity/extent of endothelial dysfunction and supporting the integrity of evoked vasodilation.

### Atherosclerosis

Endothelial dysfunction is an early marker for atherosclerosis in conduit arteries and can be detected before structural changes to the vessel wall are apparent upon angiography or ultrasound analysis. Many of the risk factors that predispose arteries to atherosclerosis can also cause endothelial dysfunction, and the presence of multiple risk factors has been found to predict endothelial dysfunction [63,75]. Under atherosclerotic conditions (e.g. hyperlipidemia, metabolic syndrome), endothelial dysfunction characterised by reduced NO bioavailability and reduced responsiveness to exogenous vasodilators, is now recognised as an early, reversible precursor of atherosclerosis [76]. While the pathogenesis of endothelial dysfunction is multifactorial, elevated oxidative stress appears to be a frequent underlying cellular driver in the ensuing loss of vasoactive, inflammatory, haemostatic and redox homeostasis in the vascular system. Endothelial dysfunction is an important factor contributing to plaque formation and disease progression in conduit arteries, and is further associated with impaired endothelium-dependent relaxation and increased levels of reactive oxygen species [56]. Upregulation of NO production or activity and reduction of oxidative stress decrease the likelihood of plaque formation and atherosclerosis [56,77]. We have previously reported that acute administration of SKA-31 elicits robust vasodilation of the coronary circulation in isolated, beating male and female rat hearts [48], and this effect is preserved in hearts from non-obese, T2D rats [5].

Whereas larger doses of an endothelial KCa channel activator elicit acute hypotensive responses, low circulating levels of SKA-31 or a comparable activator in the vasculature may be anticipated to enhance agonist-evoked vasodilation via EDH and NO-related signaling pathways, as we observed in isolated beating hearts [5]. Pharmacologic “priming” or sensitization of endothelial KCa channel activity may thus promote vasculature health by opposing pro-atherosclerotic processes and aberrant vascular wall remodelling, as we have previously discussed [6]. Interestingly, and somewhat unexpectedly, Bi et al. (2013) have reported that KCa3.1 activation with channel agonists such as SKA-31, NS309 or EBIO was able to block PDGF-stimulated proliferation of coronary artery smooth muscle cells *in vitro* more effectively than the KCa3.1 channel inhibitor TRAM-34 [78]. Mechanistically, these agonists decreased KCa3.1 expression in proliferative smooth muscle cells, thereby dampening Ca^2+^ mobilization, Ca^2+^-dependent signaling pathways and cell cycle progression. Reduced vascular smooth muscle cell hyperplasia/hypertrophy would be expected to decrease plaque formation/size in atherosclerosis. Whether *in vivo* administration of a KCa3.1 channel agonist similarly inhibits vascular smooth cell proliferation in an animal model of atherosclerosis is unknown. Collectively, these observations suggest that KCa3.1 channel activators, such as SKA-31, may provide novel therapeutic benefit for diseases characterized by increased cell proliferative activity, such as atherosclerosis [78].

### Ageing

Ageing directly contributes to the onset and severity of cardiovascular disease (CVD) [58], and it is now recognized that mitigating age-related endothelial dysfunction should improve the health of aged patients with CVD [58]. Endothelial dysfunction due to age is associated with compromised bioavailability of NO and altered voltage-gated Ca^2+^ channel signalling pathways [79]. EDH initiated through KCa channel activation represents an important vasodilatory mechanism in resistance vessels and alterations in this signalling pathway have been associated with vascular disease in ageing [80]. Although several factors can predispose to endothelial dysfunction, it is now evident that endothelial oxidative stress increases with age, which can lead to coronary artery disease. In the presence of elevated oxidative stress, NO + O_2_^−^ readily combine to form peroxynitrite (ONOO^−^), thereby reducing NO bioavailability and the ability to maintain vascular homeostasis [81,82]. Chennupati and colleagues have reported that the contribution of EDH to endothelium-dependent relaxations in conduit arteries modestly decreases with age, whereas the contribution of endothelium-derived NO slightly increases in old male mice [83]. Related studies from Behringer et al. (2013) have reported that upregulation of endothelial KCa channel activity in small resistance arteries from skeletal muscle of aged mice impairs the longitudinal spread of endothelium-derived hyperpolarization that supports conducted vasodilation along the vascular network and the regulation of local blood flow [83].

## Summary

Small molecule activators of endothelial KCa2.3 and KCa3.1 channels may represent a novel therapeutic strategy in the treatment of a variety of CVD conditions and other pathologies, as highlighted by Wulff, Köhler and colleagues [3,15,38,85]. SKA-31, a second generation activator of KCa2.3 and KCa3.1 channels, has proven valuable as a pharmacological tool to investigate endothelium-dependent vasodilatory processes and represents an attractive prototypic compound for translational studies in whole tissues and animals. Additional pharmacokinetic and toxicologic data in whole animals and pre-clinical models are needed to establish the therapeutic applicability and effectiveness of SKA-31 and related compounds in cardiovascular pathologies. Progress in these areas will help determine the therapeutic potential of this class of drug in the treatment of endothelium/vascular-related disorders.

## Abbreviations

*cGMP*: cyclic guanosine 3’-5’-monophosphate
*EDH*: endothelial-derived hyperpolarization
*eNOS*: endothelial nitric oxide synthase
*GPCR*: G-protein-coupled receptor
*GSK101*: GSK1016790A
*KCa2.3*: small conductance, Ca^2+^-activated K^+^ channel 2.3
*KCa3.1*: intermediate conductance, Ca^2+^-activated K^+^ channel 3.1
*MEGJ*: myo-endothelial gap junction
*NO*: nitric oxide; PKG, protein kinase G
*IP_3_*: inositol 1,4,5-trisphosphate
*PKG*: protein kinase G
*PLC-β*: phospholipase C-beta
*sGC*: soluble guanylyl cyclase
*SOCE*: store-operated Ca^2+^ entry
*VGCC*: voltage-gated Ca^2+^ channel
